# Distinct TP53 Mutation Subtypes Differentially Influence Cellular Iron Metabolism

**DOI:** 10.3390/nu11092144

**Published:** 2019-09-07

**Authors:** Stephen L. Clarke, Laurie R. Thompson, Eshan Dandekar, Aishwarya Srinivasan, McKale R. Montgomery

**Affiliations:** Department of Nutritional Sciences, Oklahoma State University, Stillwater, OK 74074, USAmckale.montgomery@okstate.edu (M.R.M.)

**Keywords:** mutant TP53, metabolism, cancer, iron regulator proteins, iron-sulfur cluster biogenesis

## Abstract

The most commonly mutated gene in all human cancers is the tumor suppressor gene *TP53*; however, in addition to the loss of tumor suppressor functions, mutations in *TP53* can also promote cancer progression by altering cellular iron acquisition and metabolism. The primary objective of this work was to determine how *TP53* mutation status influences the molecular control of iron homeostasis. The effect of *TP53* mutation type on cellular iron homeostasis was examined using cell lines with inducible versions of either wild-type *TP53* or a representative mutated *TP53* gene from exemplary “hotspot” mutations in the DNA binding domain (R248, R273, and R175) as well as H193Y. The introduction of distinct TP53 mutation types alone was sufficient to disrupt cellular iron metabolism. These effects were mediated, at least in part, due to differences in the responsiveness of iron regulatory proteins (IRPs) to cellular iron availability. IRPs are considered the master regulators of intracellular iron homeostasis because they coordinate the expression of iron storage (ferritin) and iron uptake (transferrin receptor) genes. In response to changes in iron availability, cells harboring either a wild-type TP53 or R273H TP53 mutation displayed canonical IRP-mediated responses, but neither IRP1 RNA binding activity nor IRP2 protein levels were affected by changes in iron status in cells harboring the R175H mutation type. However, all mutation types exhibited robust changes in ferritin and transferrin receptor protein expression in response to iron loading and iron chelation, respectively. These findings suggest a novel, IRP-independent mode of iron regulation in cells expressing distinct TP53 mutations. As *TP53* is mutated in nearly half of all human cancers, and iron is necessary for cancer cell growth and proliferation, the studies have implications for a wide range of clinically important cancers.

## 1. Introduction

The essentiality of iron for cell growth and proliferation, coupled with its capacity to promote damaging free radical production, has made it a desirable target for cancer treatment and prevention. Indeed, distinct iron-related gene expression patterns are associated with both the development of metastases and patient survival [1,2,3,4]. However, it is important to first understand how cancer cells manipulate the homeostatic regulators of iron metabolism to promote malignancy before we can fully harness iron’s therapeutic potential. One such mechanism may be through the acquisition of *TP53* mutations. For example, cells that lack TP53, or express a mutant TP53, accumulate iron in response to DNA damage [5]. Mutations in TP53 have also been shown to decrease tumor cell responsiveness to iron restriction [6]. However, the inactivation of iron regulatory proteins can facilitate wild-type TP53-mediated cell cycle arrest [7], and the consequential effects of distinct TP53 mutation types on cellular iron metabolism remain unknown.

Intracellular iron homeostasis is regulated by two cytosolic mRNA binding proteins, iron regulatory protein (IRP)1 and IRP2, that function by “sensing” intracellular iron status and, accordingly, coordinate the uptake, storage, and utilization of iron. When cytosolic iron levels are limited, IRPs bind to iron responsive elements (IREs) with high affinity. High-affinity RNA binding results in the inhibition of the translation of mRNA containing 5′ IRE, such as h-ferritin (FTH1) and the stabilization of mRNA containing 3′IRE, such as transferrin receptor (TFRC) [8]. Under iron replete conditions, IRPs lose their high-affinity RNA binding activity. IRP2 levels are controlled by iron-dependent proteasomal degradation. IRP1 is regulated via the formation of an iron–sulfur (Fe–S) cluster, which inhibits its RNA binding activity [9].

The capacity for TP53 to contribute to iron homeostasis was first recognized by Zhang et al., who demonstrated that TP53-dependent growth arrest is facilitated by restricting cellular iron availability via the inactivation of the IRE–IRP system [7]. The connection between TP53 and IRP regulation was recently expanded upon by the discovery that wild-type TP53 specifically modulates IRP1 RNA binding activity via the transcriptional regulation of the Fe–S cluster assembly enzyme (ISCU) [5]. This study also demonstrated that excess dietary iron significantly increases serum iron levels in TP53 knockout mice, but not in mice expressing wild-type TP53. Moreover, this work established that decreased ISCU expression in human liver cancer tissues is associated with TP53 mutation. However, the impact of TP53 mutation status on IRP RNA binding activity and the control of cellular iron homeostasis has not been investigated.

Although hundreds of TP53 mutations have been identified, the majority occur within the DNA binding domain and can be subdivided into two broad classes: contact or conformational. Mutations are categorized as “contact” when they occur in regions that make direct contact with DNA sequences, and “conformational” when they disrupt the structure of the TP53 protein itself [10]. These are functionally important distinctions, because mutation type significantly impacts mutant TP53 binding partners [10,11,12,13]. In the present study, we demonstrate that the introduction of distinct TP53 mutation types alone is sufficient to significantly and differentially alter total cellular iron levels as well as spontaneous IRP RNA binding activity. Moreover, we also show that cells harboring distinct TP53 mutation types exhibit differential responses to changes in iron availability. Taken together, these results illustrate that TP53 mutation status can significantly influence the control of cellular iron homeostasis.

## 2. Materials and Methods 

### 2.1. Cell Culture Conditions and Creation of Inducible Cell Lines

H1299 cells, which are null for TP53, were obtained from the American Type Culture Collection (CRL-5803) and maintained in RPMI-40 (Corning) containing 10% tetracycline-free FBS (Atlanta Biologicals) and 100 IU/mL penicillin and 100 (μg/mL) streptomycin in a temperature and humidity-controlled incubator. Wild-type and mutant TP53 containing pcDNA5/TO (ThermoFisher; Waltham, MA, USA) inducible expression plasmids were prepared using a custom cloning service (GenScript; Piscataway, NJ, USA) and validated by Sanger sequencing to authenticate the mutations and ensure quality of the construct. To obtain stable, tetracycline-inducible TP53 expressing cell lines, H1299 cells were co-transfected with the inducible expression plasmids and pcDNA6/TR (ThermoFisher; Waltham, MA, USA) followed by selection with 10 µg/mL blasticidin and 600 µg/mL hygromycin. In total, six cell lines were generated. H1299 cells expressing only the pcDNA6/TR and empty pcDNA5/TO vectors (H1299) were used as TP53 null control cells. The five other cell lines expressed both pcDNA6/TR and either a wild-type *TP53* gene (WT), or a representative mutated *TP53* gene from exemplary “hotspot” mutations in the DNA binding domain (R248Q, R273H, and R175H) as well as H193Y. These mutations were selected because they represent the most common examples of TP53 contact (R248Q and R273H) and conformational (R175H) mutants. The H193Y mutant was included because we had previously observed that shRNA knockdown of TP53 in cells expressing an endogenous TP53 mutation was sufficient to alter iron-related gene expression and increase cellular iron levels (data not shown), so we wanted to further examine its potential role in the regulation of cellular iron homeostasis. The expression of TP53 was induced by treating cells with 10 µg/mL tetracycline for 24 h. The efficacy of tetracycline-dependent repression and induction was confirmed by Western blot. To examine the influence of TP53 expression on the response to changes in iron availability, cells were simultaneously induced with tetracycline and treated with 40 µM hemin or 50 µM desferrioxamine (DFO) for 24–48 h to induce iron overload or reduce iron availability, respectively. 

### 2.2. Western Blots

Total protein was collected from cells following lysis with radioimmunoprecipitation assay (RIPA) buffer (50 mM Tris-HCL, pH 8.0, 1% NP-40, 0.5% Na-deoxycholate, 0.1% SDS, 2 mM EDTA, 150 mM NaCl) supplemented with Halt Protease and Phosphatase Inhibitor Cocktail (ThermoFisher; Waltham, MA, USA), 1 mM DTT, 1 mM citrate, 0.1 mM phenylmethylsulfonyl fluoride, and 10 µM MG-132 and with centrifugation at 14,000× *g* for 20 min. Following the determination of protein concentration by bicinchoninic acid assay (ThermoFisher; Waltham, MA, USA), 30 µg total protein per well was loaded into a 4–20% tris-glycine precast gel (Bio-Rad) and electrophoresed at 150 volts for 1 h before being transferred to a PVDF membrane. FTH1 (4393S), TFRC (13208S), and TP53 (2425S) antibodies were obtained from Cell Signaling Technology (Danvers, MA, USA). Anti-FDXR (ab204310) was purchased from Abcam (Cambridge, MA, USA). IRP1 and IRP2 antibodies were generous gifts from Dr. Rick Eisenstein (University of Wisconsin). Anti-gamma-tubulin (T6557) was purchased from Sigma-Aldrich. Blots were incubated in primary antibodies overnight at 4 °C at a 1:1000 dilution in 5% non-fat dry milk in 1X tris-buffered saline with 0.01% tween-20. HRP-linked secondary antibodies were used at a 1:10,000 dilution to detect primary antibody binding using chemiluminescence (ECL; GE Healthcare). Blots were imaged using the ProteinSimple Fluorhem R (R&D Systems; Minneapolis, MN, USA) and analyzed using ImageJ software (NIH) [14]. First, the optical density of individual protein bands was plotted and quantified using the analyze gels function in ImageJ. Relative abundance was then quantified by calculating individual band as a percent of total protein band density followed by normalization to anti-gamma-tubulin (TUBG1) band density to control for differences in loading. 

### 2.3. Intracellular Iron Measurement

Following induction with 10 µg/mL tetracycline for 24 h, at least 1 × 10^6^ cells were lysed, and total intracellular iron levels were determined by measuring its colorimetric reaction with chromagen using an iron assay kit (MilliporeSigma; Burlington, MA, USA). Total protein concentrations were coordinately determined by bicinchoninic acid assay (ThermoFisher; Waltham, MA, USA), meaning that total intracellular iron levels could be normalized to nmol total iron per mg total protein. 

### 2.4. RNA Isolation and Real-Time qPCR

To analyze changes in mRNA expression following the induction of TP53 expression and/or treatment with hemin or DFO, total RNA was isolated using TRIzol reagent (ThermoFisher; Waltham, MA, USA). RNA purity and integrity were confirmed by Nanodrop (ThermoFisher) and agarose gel electrophoresis, respectively, before reverse transcription using SuperScript II (Invitrogen). The relative abundance of cyclin dependent kinase inhibitor 1A (*CDKN1*), *ISCU1/2*, *TFRC*, N-MYC downstream regulated gene 1 (*NDRG1*), and heme oxygenase 1 (*HMOX1*) were determined by qPCR using SYBR green chemistry on an ABI 7900HT Real-Time PCR system (ThermoFisher; Waltham, MA, USA) and normalized relative to peptidylprolyl isomerase B (*PPIB*) abundance using the 2^−ΔΔCt^ method. *CDKN1A*, *ISCU*, and *TRFC* primer sequences were obtained from previously published sources [5,15,16]. *PPIB* (forward—5′tgccatcgccaaggagtag; reverse—5′tgcacagacggtcactcaaa), *NDRG1* (forward—5′gtggagggccttgtccttatc; reverse—5′aggcggcccagtccat), and *HMOX1* (forward—5′cgggccagcaacaaagtg; reverse—5′agtgtaaggacccatcggagaa), with primer sequences designed using Primer Express v3.0 software and validated by both template titration and dissociation curves in addition to exhibiting an efficiency slope of −3.3.

### 2.5. Aconitase Assays

Cytosolic and mitochondrial aconitase assays were measured using an Aconitase Activity Assay kit (MilliporeSigma; Burlington, MA, USA) that determines aconitase activity using a coupled reaction in which citrate is converted to isocitrate by aconitase. The amount of isocitrate generated was normalized to the total amount of protein in a given sample so that aconitase activity could be reported in milliunit activity per milligram protein. 

### 2.6. IRP RNA Binding Assays

IRP RNA binding activity was determined by gel-shift analysis as previously described [17]. Briefly, cytosolic protein was collected by lysing cells in two-volume cytosol buffer (1 M HEPES 10 mM, 10 mM KCl, 1%, 0.1 mM EGTA, 0.1 mM EDTA) supplemented with 1 mM citrate, 1 mM DTT, 0.1 M PMSF, and 100X Halt Protease and phosphatase inhibitor cocktail (ThermoFisher; Waltham, MA, USA). Following 15 min of incubation on ice, NP40 was added to a final volume of 1%. Samples were vortexed vigorously for 10 s, then centrifuged at 12,000× *g* for 10 min at 4 °C. The supernatant (cytosol) was then collected, and the concentration was determined by bicinchoninic acid assay. Spontaneous IRP1 and IRP2 RNA binding activities were measured after incubating 5 µg cytosolic protein with saturating levels of (^32^P) labeled RNA from the L-ferritin IRE. RNA binding activity was quantified using Optiquant Acquisition and Analysis software (Packard Bioscience) and are expressed as fmol RNA bound/mg protein.

### 2.7. MTT Assays

Cells were plated at 5 × 10^3^ cells per well in 96-well plates and incubated with DMSO (control), or 0.5, 0.75, or 1 µM doxorubicin (Selleck Chem; Houston, TX, USA) for 24 or 48 h. Following treatment, 10 µL of 12 mM MTT was added to each well, and cells were placed back in the incubator at 37 °C for 4 h. The formazan was solubilized by adding 100 µL solubilization solution (40% DMSO, 16% SDS, 2% acetic acid) followed by another incubation at 37 °C for 1 h. Spectrophotometric absorbance at 570 nm was measured with a microplate reader (Synergy HT, Biotek; Winooski, VT, USA). Each assay included four technical replicates and was repeated at least three times. To account for differences in NADPH production following the induction of TP53 expression, results for each mutation were normalized relative to their own DMSO-treated controls.

### 2.8. Statistical Analyses

Differences between cell lines following the induction of TP53 expression were assessed by one-way ANOVA. Differences between cell lines before and after iron or doxorubicin treatments were analyzed using a two-factor mixed design ANOVA, followed by Bonferroni correction to adjust for multiple pairwise comparisons. Student’s t-tests were used to determine differences relative to controls within a given cell type. All tests were performed using SPSS v23.0 software (IBM-SPSS; Chicago, IL, USA). Differences were considered statistically significant at the 95% confidence level (alpha = 0.05). Descriptive statistics were calculated for all variables and include mean ± SEM. 

## 3. Results

### 3.1. Induction of Mutant TP53 Expression Alone is Sufficient to Alter Cellular Iron Levels

**Figure 1:**
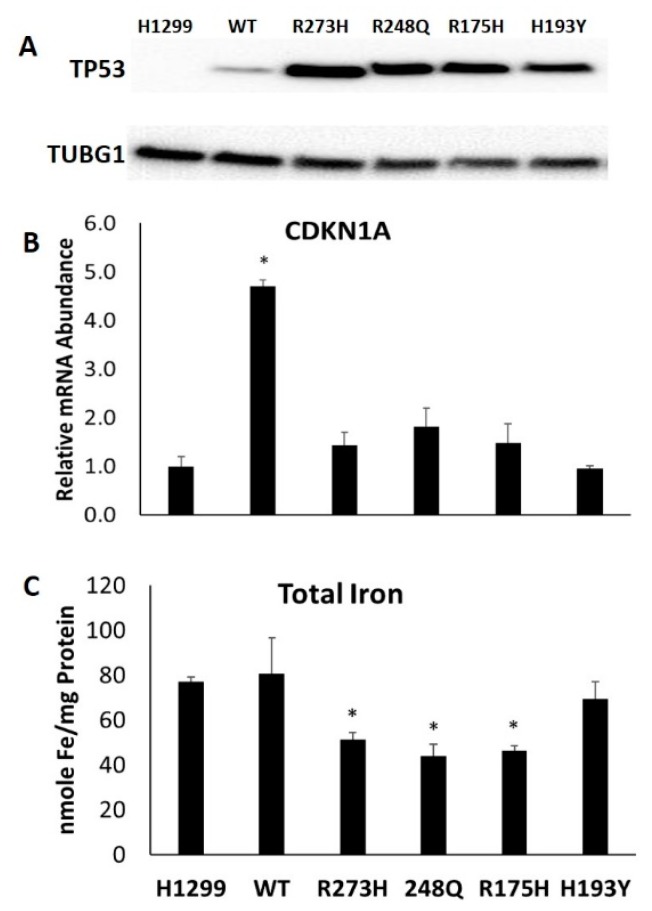
Influence of TP53 induction on total intracellular iron levels. TP53 null H1299 cells (H1299) were transfected with either a tetracycline inducible wild-type (WT) TP53 or a representative contact (R237H or R248Q) or conformational (R175H or H193Y) mutant TP53. (**A**) Western blot of TP53 expression levels in each following tetracycline induction. (**B**) Relative *CDKNA1A* mRNA expression levels following tetracycline induction of WT TP53 and indicated TP53 mutants. (**C**) Total intracellular iron was differentially altered in response to the induction of distinct TP53 mutation types. * Denotes statistical difference from H1299, *p* < 0.05.

To determine the importance of TP53 mutation status on cellular iron homeostasis, H1299 cells were generated that could express wild-type or mutant TP53 in an inducible manner. Treatment with 10 µg/mL tetracycline for 24 h was sufficient to induce the wild-type and mutant TP53 protein expression in H1299 cells (Figure 1A). TP53 expression levels were variable amongst WT and mutant TP53-expressing subtypes, with WT cells exhibiting the lowest levels of TP53 protein abundance. However, this level of induction was still sufficient to induce the mRNA expression of the WT TP53 target CDKN1A (Figure 1B). Consistent with previous reports, CDKN1A expression was not induced by any of the mutant TP53 expressing cell lines tested [18], but total intracellular iron levels were reduced in R273H, R248Q, and R175H subtypes (Figure 1C). These results indicate that TP53 mutation status can significantly influence the control of cellular iron abundance.

### 3.2. Iron-Related mRNA Expresion Differs among Cells Harboring Distinct TP53 Mutation Subtypes

**Figure 2:**
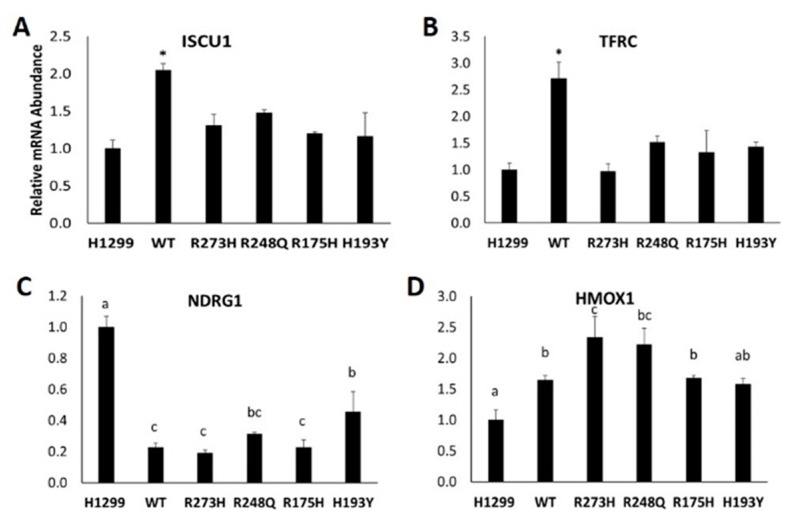
Effect of mutant TP53 expression on iron-related gene expression. Relative mRNA expression of (**A**) iron–sulfur cluster assembly enzyme 1 (ISCU1), (**B**) transferrin receptor 1 (TFRC), (**C**) N-MYC downstream regulated gene 1 (NDRG1), and (**D**) heme oxygenase 1 (HMOX1) in TP53 null H1299 cells (H1299) or H1299 cells transfected with wild-type (WT) TP53, or the indicated mutant TP53. * Denotes statistical difference from H1299. Superscripts (a,b,c) denote statistical significance between mutation types, *p* < 0.05. Treatments that share the same superscripts are not significantly different.

In agreement with others [5], the induction of WT TP53 expression alone is sufficient to significantly increase *ISCU* mRNA expression (Figure 2A). *TFRC* mRNA expression also significantly increased with the induction of WT TP53 expression (Figure 2B) compared to the TP53 null (H1299) cell line. We next examined the expression of other previously identified iron-related TP53 targets including HMOX1 and NDRG1, which are essential for heme catabolism and TP53-dependent apoptosis, respectively [19,20]. Contrary to previous findings, *NDRG1* mRNA expression was decreased following the induction of WT TP53 and each of the mutants tested (Figure 2C). The induction of both WT and mutant TP53 expression significantly increased *HMOX1* mRNA expression (Figure 2D).

**Figure 3:**
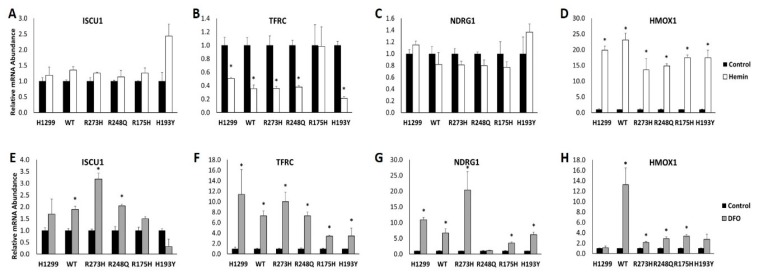
Impact of mutant TP53 expression on iron-dependent changes in gene expression. Relative mRNA expression of (**A**) iron–sulfur cluster assembly enzyme 1 (ISCU1), (**B**) transferrin receptor 1 (TFRC), (**C**) NMYC downstream regulated gene 1 (NDRG1), and (**D**) heme oxygenase 1 (HMOX1) in TP53 null H1299 cells (H1299) or H1299 cells transfected with wild-type (WT) TP53, or the indicated mutant TP53 following treatment with DMSO (control) or 40 µM hemin for 48 h. Relative mRNA expression of (**E**) ISCU1, (**F**) TFRC, (**G**) NDRG1, and (**H**) HMOX1 in the cells described above following treatment with DMSO (control) or 50 µM desferrioxamine (DFO) for 48 h. * Denotes statistical significance from respective controls, *p* < 0.05.

To investigate whether TP53 expression status would influence the molecular response to changes in iron availability, cells were treated with hemin to induce iron overload or a potent iron chelator (DFO) to reduce cellular iron availability. *ISCU* and *NDRG1* mRNA expression were not influenced by hemin treatment in any of the cell types tested (Figure 3A,C), while *HMOX1* expression was increased in all cell types in response to iron loading (Figure 3D). *TFRC* mRNA abundance was assessed as a positive control as its expression should decrease in response to iron excess as result of IRP binding to IRE within its 3′untranslated region [21]. As expected, *TFRC* expression was significantly decreased in hemin-treated TP53 null, WT, and mutant TP53-expressing cells compared to their untreated controls (Figure 3B). Conversely, *TFRC* mRNA expression was unaffected by hemin treatment in the R175H mutant TP53-expressing cell line (Figure 3B). 

DFO treatment increased the expression of *ISCU* mRNA in the WT TP53 and mutant R273H and R248Q TP53 expressing cells, but not in the TP53 null cells or R175H or H193Y mutants (Figure 3E). Expression of the positive control, *TFRC*, was increased in response to iron chelation in all cell types, but to varying degrees (Figure 3F). The expression of the metastasis suppressor NDRG1 has also been shown to increase in response to iron chelation [22]. Consistent with these reports, DFO treatment increased *NDRG1* mRNA expression in all TP53 subtypes examined, with the exception of the R248Q mutant TP53-expressing cell line (Figure 3G). Notably, however, the degree of change was quite variable, with a near 20-fold increase in *NDRG1* expression in the R273H mutants, but only a five-fold increase in the R175H mutants. *HMOX1* mRNA expression was not changed following DFO treatment in the TP53 null cell line, but was significantly increased in the WT and mutant TP53 expressing cell lines (Figure 3H). However, the mutant TP53 expressing cells exhibited a more attenuated *HMOX1* response to iron chelation than the WT cells. 

### 3.3. Cells with Distinct TP53 Mutations Exhibit Differential Changes in IRP RNA Binding Activity in Response to Modifications in Iron Availability

**Figure 4:**
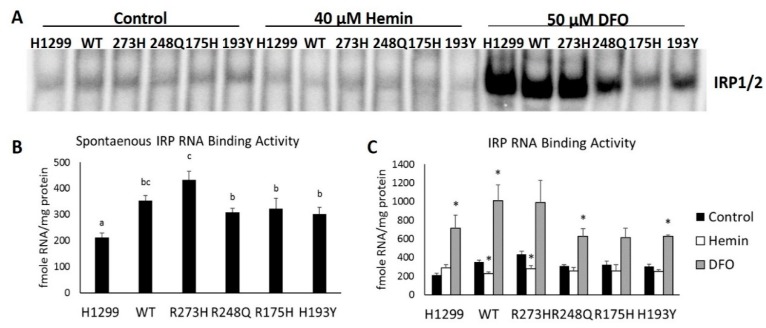
TP53-dependent responsiveness of IRP RNA binding activity following under normal, high, and low iron conditions. Spontaneous IRP RNA binding was assayed following tetracycline induction of WT or the indicated TP53 mutants under control conditions (**A**,**B**) or following treatment with 40 µM hemin or 50 µM desferrioxamine (DFO) for 48 h (**A**,**C**). Superscripts (a,b,c) denote statistical difference between mutation types. * Denotes statistical difference from respective controls, *p* < 0.05.

IRPs are considered the master regulators of cellular iron homeostasis because they coordinate the expression of genes that regulate iron uptake (TFRC) and iron storage (FTH1) in response to both low and high iron conditions. Both IRP1 and IRP2 regulation has also been linked to changes in WT TP53 protein expression [5,7,23], but the impact of mutant TP53 expression on IRP RNA binding activity has not been thoroughly investigated. Human IRP1 and IRP2 do not separate during standard gel-shift analyses [24]; thus, in this study, changes in IRP RNA binding activity refer to the sum of RNA-bound IRP1 and IRP2. Here, we demonstrate that the introduction of distinct TP53 mutation types alone is sufficient to significantly and differentially alter spontaneous IRP RNA binding activity (Figure 4A,B). Moreover, we also show that cells harboring distinct TP53 mutation types exhibit differential responses to changes in iron availability (Figure 4A,C). As predicted, IRP RNA binding activity decreases in WT and the contact (R273H) mutant TP53-expressing cells in response to excess iron. However, IRP RNA binding activity is unchanged following hemin treatment in TP53 null cells and in cells harboring R248Q, R175H, and H193Y TP53 mutations. In response to iron chelation, IRP RNA binding activity increases in TP53 null cells, as well as WT TP53, R248Q, and H193Y-expressing mutants, but is not statistically differentially altered in conformational R175H-expressing mutants (Figure 4C). These results demonstrate that distinct TP53 mutation types differentially influences IRP RNA binding activity. They also show that the TP53 mutation type can significantly alter IRP responsiveness to changes in cellular iron availability.

**Figure 5:**
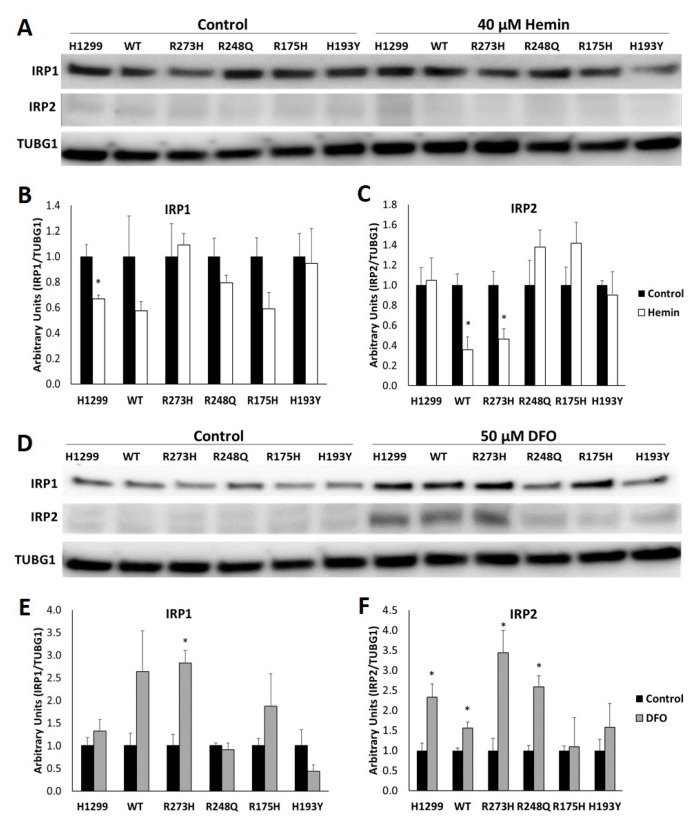
TP53 expression influences the iron-dependent regulation of IRP1 and IRP2 protein expression. Representative Western blots of IRP1 and IRP2 protein expression in TP53 null H1299 cells (H1299) or following the induction of the indicated TP53 subtype and treatment with 40 µM hemin (**A**) or 50 µM DFO (**D**) for 24 h. Relative expression levels were quantitated following normalization to anti-gamma-tubulin (TUBG1) as the loading control (**B**,**C**,**E**,**F**). * Denotes statistical difference from respective controls, *p* < 0.05.

Because human IRP1 and IRP2 do not separate during standard gel-shift analyses, we also examined total protein expression of each IRP independently via Western blot. Although the dominant mechanism for IRP1 regulation by iron is through the assembly or disassembly of an [4Fe-4S], regulation through protein degradation does occur [25,26,27]. In response to changes in iron availability, IRP1 protein expression was only statistically significantly decreased following hemin treatment in the TP53 null cell line (Figure 5A,B) and significantly increased following iron chelation in the R273H cell line (Figure 5E). Contrary to IRP1, IRP2 is primarily regulated at the level of protein stability. When iron or oxygen are limited, IRP2 is stabilized, but in the presence of iron and oxygen, IRP2 is targeted for proteasomal degradation [26,27]. Remarkably, in response to hemin treatment, IRP2 was only significantly decreased in the WT TP53 and R273H TP53 mutant cell lines compared to their untreated controls, but was unchanged in the other cell lines tested. Iron restriction significantly increased IRP2 expression in TP53 null, WT TP53, R273H and R248Q-expressing mutant cells, but did not affect IRP2 protein levels in the R175H or H193Y mutants (Figure 5B). Thus, because neither IRP1 nor IRP2 protein abundance was altered by the induction of WT or mutant TP53 expression alone, the observed TP53-dependent alterations in RNA binding in Figure 4A,B can be attributed to changes in IRP RNA binding activity and not changes in IRP abundance. Moreover, the induction of distinct TP53 mutation types may alter the iron-dependent regulation of IRP1 and IRP2 RNA binding activity by altering Fe-S cluster biogenesis activity and iron-dependent protein regulation, respectively.

### 3.4. Reduced FDXR Expression is Associated with Decreased Mitochondrial Aconitase Activity in R175H Mutants

**Figure 6:**
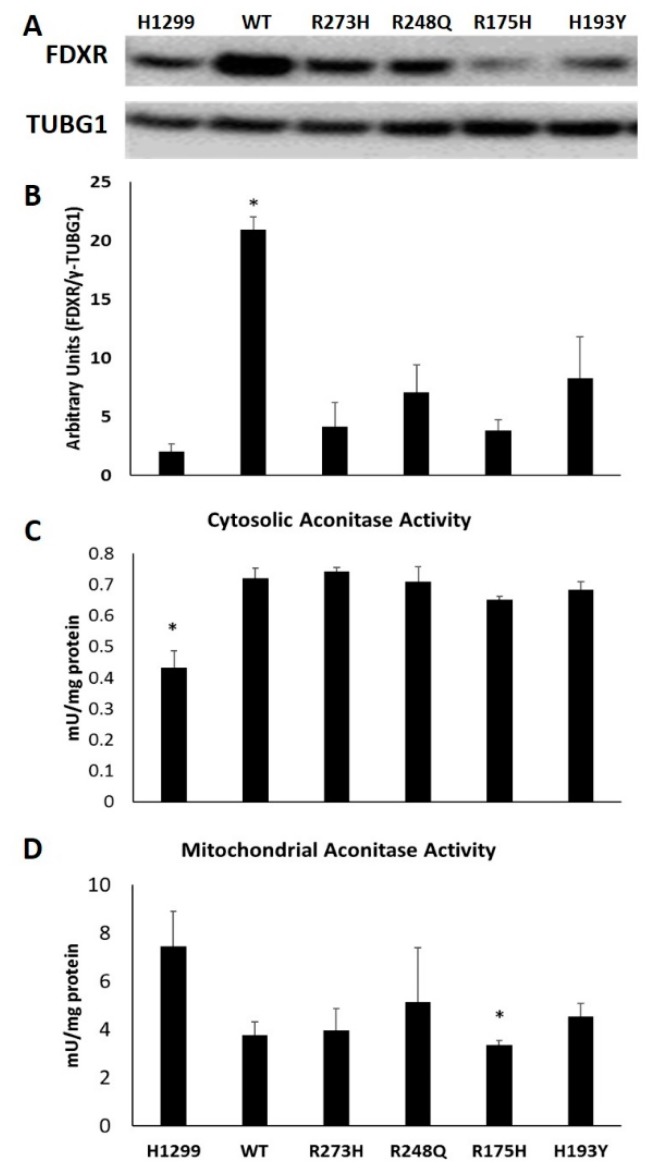
Impact of WT and mutant TP53 expression on Fe-S cluster biogenesis. Representative Western blot analysis of ferredoxin reductase (FDXR) expression in TP53 null cells (H1299) and following tetracycline induction of indicated TP53 expression types (**A**). FDXR protein quantitation following normalization to TUBG1 (**B**). Cytosolic (**C**) and mitochondrial (**D**) aconitase activity were also measured in the same cell types. * Denotes statistical difference, *p* < 0.05.

To identify potential mechanisms contributing to the lack of IRP responsiveness in the mutant cell lines, we utilized mass spectrometry to examine total differential protein expression between cells transfected with either WT TP53 or with the conformational R175H mutant, as these cells represented the most pronounced IRP-responsive phenotypes. These studies revealed that one of the most differentially expressed proteins was ferredoxin reductase (FDXR), which was decreased nearly four-fold in the R175H mutant cells (Supplemental Table S1). These findings were validated by Western blot, and similar reductions in FDXR expression was observed in all mutant TP53 expressing cell lines, with R175H mutants exhibiting the lowest levels of FDXR expression (Figure 6A,B). In humans, FDXR is critical for Fe–S cluster biogenesis, and its reduction is associated with the misregulation of cellular iron homeostasis [28]. As Fe–S assembly/disassembly is the primary determinant of IRP1 function, impaired Fe–S biogenesis could explain the observed lack of a decrease in IRP RNA binding activity in R175H mutant cells in response to hemin treatment. To examine the impact of reduced FDXR further, we measured the enzymatic activity of cytosolic aconitase (IRP1) in each of our cell lines. We also measured mitochondrial aconitase activity because it is also an Fe–S cluster-containing enzyme that exhibits reduced enzymatic activity in response to reduced FDXR expression [28]. Cytosolic aconitase activity was lower in the TP53 null cell line than any of the WT or mutant TP53-expressing cell lines tested (Figure 6C). Compared to the TP53 null line, mitochondrial aconitase had a trend towards decreased activity in WT (*p* = 0.05) and R273H (*p* = 0.06) expressing cells, but was only statistically significantly decreased in the R175H mutants (*p* = 0.03) (Figure 6D). No difference in cytosolic aconitase activity between WT and mutant TP53-expressing cells suggests that, under steady state or basal conditions, cytosolic Fe–S cluster biogenesis is not affected by reduced FDXR expression. However, mitochondrial aconitase activity was significantly decreased in the R175H mutant cell line, which also had the lowest level of FDXR expression.

### 3.5. Mutant TP53 Expressing Cells Display Robust FTH1 and TFRC Responses to Changes in Iron Availability

**Figure 7:**
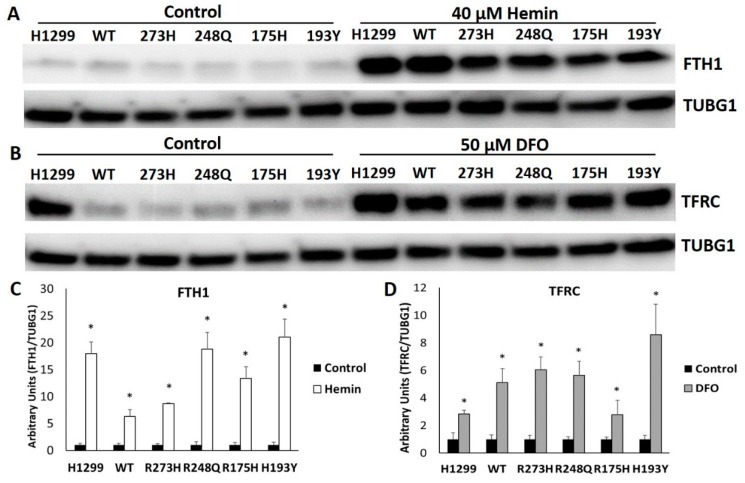
Iron-dependent changes in FTH1 and TFRC protein expression in mutant TP53 expressing cells. Representative Western blot of FTH1 expression following treatment with 50 µM hemin (**A**) and TFRC expression following treatment with 40 µM DFO (**B**) protein expression in TP53 null H1299 cells (H1299) or following the induction of the indicated TP53 subtype (**A**). Relative expression levels were quantitated following normalization to TUBG1 as the loading control (**C**,**D**). * Denotes statistical difference from respective controls, *p* < 0.05.

Increased IRP RNA binding activity in response to decreased iron availability ultimately results in increased iron uptake via the increased expression of TFRC and the release of stored iron by degrading FTH1 [8]. In response to iron excess, IRP RNA binding activity decreases. Consequently, TFRC expression and iron uptake are reduced, and the uptake of iron into FTH1 is enhanced. As neither IRP RNA binding activity nor IRP2 protein was decreased in response to hemin treatment in cells null for TP53 or in R248Q, R175H, H193Y-expressing mutants, we predicted FTH1 expression would not be changed in these cell types either. However, all cell types tested displayed significant increases in FTH1 expression in response to hemin treatment (Figure 7A,B). Moreover, despite the lack of a change in IRP RNA binding activity in the R175H mutants and a lack of IRP2 responsiveness to iron chelation in both the R175H and H193Y mutants, all TP53-expressing subtypes tested also displayed considerable increases in TFRC expression in response to DFO treatment (Figure 7C,D). As FTH1 and TFRC are canonically regulated by iron-dependent changes in IRP RNA binding activity, these results suggest a novel mode of IRP-independent regulation in these cell types.

## 4. Discussion

The tumor suppressor TP53 is the most commonly mutated gene in human cancers, and iron is necessary for cancer cell growth and proliferation, but there is a paucity of data delineating the contribution of TP53 mutations to the regulation of iron homeostasis. There are even less data to show how iron availability influences therapeutic sensitivity in cells with different types of TP53 mutations. To date, the targeting of mutant TP53 has primarily focused on restoring its wild-type activity or promoting its degradation, while iron chelation has been a primary emphasis for the development of iron-based chemotherapy [29,30,31]. However, even the efficacy of iron deprivation is at least somewhat dependent upon wild-type TP53 signaling [6]. In this study, we have utilized an inducible expression system to characterize the influence of individual mutant TP53 proteins on the control of cellular iron homeostasis. We found that the induction of mutant TP53 expression alone is sufficient to significantly alter cellular iron levels and that distinct TP53 mutation types differentially alter the cellular response to changes in iron availability.

Wild-type TP53 has previously been demonstrated to contribute to cell cycle arrest by decreasing IRP RNA binding activity and subsequently decreasing cellular iron availability [7]. This effect may be attributable to the TP53-mediated transcriptional upregulation of ISCU expression, and consequently increase in Fe–S biogenesis [5]. Consistent with these findings, we also observed a significant two-fold increase in ISCU mRNA expression in response to the induction of WT TP53, but not with any of the mutant TP53 proteins tested. Similar to previous observations, we also detected a significant increase in HMOX1 with WT TP53 induction [19]. TP53-mediated regulation of HMOX1 is postulated to promote cell survival in certain contexts. We observed that the induction of mutant TP53 expression significantly increases HMOX1 mRNA expression compared to TP53 null cells as well, but the functional consequences of mutant TP53-mediated increases in HMOX1 expression are unclear. In contrast to previous findings, the expression of the iron and TP53-regulated target NDRG1 was unexpectedly decreased upon induction of WT and mutant TP53 expression. However, endogenous NDRG1 expression is near the lower limits of detection in H1299 cells under normal circumstances, and previous reports indicate this may be a cell-type specific effect [20]. These results indicate that both WT and mutant TP53 expression can influence iron-related gene expression.

Iron excess is strongly associated with tumorigenesis [32,33,34,35], and iron deprivation has demonstrated chemotherapeutic effects [36,37,38,39], but the influence of TP53 mutation status on cellular responsiveness to iron availability is less understood. We observed no changes in ISCU expression with hemin treatment, but ISCU mRNA expression was increased in WT TP53, R273H, and R248Q-expressing mutants following DFO treatment. ISCU expression has previously been reported to be decreased following DFO treatment as a result of its hypoxia mimetic effects [40]. However, DFO treatment has also been demonstrated to stabilize WT TP53 in a hypoxia-independent manner [6]. Thus, these findings may be attributable to a TP53 subtype-dependent DFO effect. The lack of a hemin-mediated effect on TFRC expression in R175H mutant-expressing cells was unanticipated, but TFRC can be regulated at multiple levels beyond transcript degradation [41], and changes in protein expression were observed, indicating these cells still respond to alterations in iron status. 

Hemin did not have an effect on NDRG1 expression in any of the cell types examined, but DFO treatment significantly increased NDRG1 expression in all cell types except the R248Q mutants. Previous reports indicated that NDRG1 expression is increased by DFO independently of TP53 status, but not all mutation types examined in the current study were previously tested [22]. As NDRG1 is an established tumor growth and metastasis suppressor, iron chelators have been posited as potential inhibitors of metastatic spread [42]. However, our findings indicate that their efficacy in this regard may be dependent upon mutant TP53 status. 

HMOX1 expression increased in response to hemin treatment regardless of TP53 status. This observation is in accordance with canonical transcriptional de-repression of HMOX1 in the presence of heme [43]. An increase in HMOX1 expression following DFO treatment in all TP53 expressing cells, however, was an unexpected finding. The large (~12-fold) DFO-mediated increase in HMOX1 expression in WT TP53 expressing cells compared to the blunted (~2–3-fold) increase in mutant TP53 expressing cells and no change in the null cells may indicate differences in how cells with distinct TP53 expression statuses adapt to iron deprivation. Indeed, previous work has demonstrated that the tumor suppressive effects of iron deprivation are dependent upon WT TP53 signaling [6]. These results, coupled with the muted NDRG1 response in R248Q mutants, suggest that TP53 mutation status may be a predictor of the efficacy of iron deprivation-based chemotherapy. Based on these findings, we hypothesize that increased HMOX1 expression following iron chelation could serve as an adaptive response of the cell in an attempt to liberate heme-bound iron for use by the cell when other iron sources are limited; however, further investigation is warranted.

Because ISCU expression was only influenced by the induction of WT TP53, and mutant TP53 expressing subtypes exhibited differential responses to changes in iron availability, we next sought to determine the impact of mutant TP53 expression on IRP RNA binding activity. Previous studies in H1299 cells and the TP53 null colon cancer HCT116 cell line reported a decrease in IRP RNA binding activity upon induction of WT TP53 expression for 24 h [7]. It has been postulated that this was due to the TP53-dependent transcriptional activation of ISCU, as knockdown of ISCU was found to be sufficient to increase IRP RNA binding activity in WT TP53 expressing cells [5]. In the present study, we found that the ~two-fold increase in ISCU expression following the induction of WT TP53 was not sufficient to decrease IRP RNA binding activity. Instead, after 48 h of WT TP53 induction, we observed a modest but statistically significant increase in total IRP RNA binding activity. The discrepancy in these findings may be attributed to differences in experimental design and could indicate an adaptive cellular response to WT TP53 induction over a longer time period. Thus, after an initial insult, the induction of WT TP53 would promote the restriction of cellular iron availability, but upon recovery, cells would need to acquire additional iron to restore homeostasis. Compared to the TP53 null cells, the induction of mutant TP53 expression for 48 h increased spontaneous IRP RNA binding activity to the same level as that of WT TP53. Three of the mutants (R273H, R248Q, and R175H) also had significantly lower iron levels than the null and WT cells. Thus, it is not clear whether the increased IRP RNA binding activity is in response to mutant TP53 signaling or an indirect response to decreased cellular iron. 

Another key finding in this study is that TP53 mutation status significantly impacts the IRP response to changes in cellular iron availability. While cells expressing WT TP53 appropriately decrease and increase IRP RNA binding activity with iron excess and iron chelation, respectively, null and mutant TP53-expressing cells exhibit abrogated responses. Besides the WT TP53-expressing cells, only the R273H-expressing mutants displayed decreased IRP RNA binding activity in response to hemin treatment. However, neither the R273H-expressing cells nor the R175H-expressing cells achieved a statistically significant increase in IRP RNA binding activity following DFO treatment—*p* = 0.08 and *p* = 0.06, respectively. These data indicate that mutant TP53 expression reduces IRP sensitivity to alterations in iron availability.

Because human IRP1 and IRP2 do not separate during standard gel-shift analyses, the effects of mutant TP53 expression on the relative contributions of IRP1 versus IRP2 to IRE binding cannot be determined based on gel-shift results alone. Therefore, we also looked at total protein expression of IRP1 and IRP2 following iron loading and iron chelation. The cytosolic aconitase pool of IRP1 often greatly exceeds that of its RNA binding form, and thus iron-mediated changes in total protein expression are not routinely observed via Western blots [44,45]. However, we did observe a statistically significant decrease in total IRP1 protein abundance following hemin treatment in the TP53 null H1299 cell line and a significant increase in total IRP1 protein abundance following iron chelation in the R273H TP53 mutant cell line. The iron-mediated proteosomal degradation of IRP1 has been previously demonstrated in models where the Fe–S switch was disrupted [27,28,46]. Moreover, we also observed reduced cytosolic aconitase activity in the TP53 null line; thus, this could contribute to the iron-mediated reduction in IRP1 expression in this cell line. The statistically significant increase in IRP1 protein expression following iron chelation in the R273H mutant cell line was an unexpected finding. However, extremely low oxygen conditions have been reported to increase IRP1 translation [47], so this may indicate a hyperactive hypoxic response to iron chelation in R273H-expressing mutant. 

IRP2, on the other hand, is typically regulated at the level of protein degradation, via the iron-dependent regulation of its E3 ubiquitin ligase, F-Box and leucine rich repeat protein 5 (FBXL5) [27,28]. Intriguingly, hemin treatment only reduced IRP2 protein expression in the WT TP53 and R273H mutant-expressing cell lines. The WT TP53 and R273H-expressing cells also exhibited the expected increase in IRP2 protein expression following iron chelation, as did the TP53 null and R248Q-expressing cell lines. However, IRP2 was unresponsive to both iron loading and iron chelation in the conformational R175H and H193Y-expressing mutants. FBXL5 has previously been shown to be reduced in WT TP53 but not mutant TP53-expressing cells in response to irradiation [48]. Thus, it is tempting to speculate that distinct TP53 mutation types (e.g., R175H and H193Y) may interfere with the iron-mediated regulation of regulation of FBXL5. 

As WT TP53 and R175H-expressing mutants represented the most extreme variations in iron-dependent IRP responsiveness, we utilized mass spectrometry to examine differences in global protein expression between these two cell lines to begin to understand what may contribute to these phenotypic variations. FDXR was found to be significantly reduced in R175H mutants compared to the WT TP53-expressing controls, and similar results were then confirmed by Western blot in TP53 null cells and all other TP53 mutants tested. FDXR knockdown has previously been demonstrated to decrease both cytosolic and mitochondrial aconitase activity [28]. In line with these findings, the R175H-expressing mutants exhibited the lowest levels of FDXR protein expression and mitochondrial aconitase activity, but cytosolic aconitase activity was unchanged. This is consistent with previous work demonstrating that mitochondrial enzymatic activity is extremely sensitive to perturbations in cellular iron metabolism [49,50]. As mitochondrial Fe–S biogenesis proteins are essential to the maturation of cytosolic and nuclear Fe–S proteins [51], impaired mitochondrial Fe–S cluster biogenesis could contribute to the apparent lack of cluster insertion into IRP1 in the R175H mutant cell line in response to iron treatment. Future studies should investigate cytosolic and mitochondrial Fe-S protein activity under iron-replete and iron-deficient conditions in WT and mutant TP53-expressing cells to test this hypothesis.

Notably, proper FDXR signaling is also critical for the IRP2-mediated control of TP53-dependent tumor suppression [23]. Repressed FDXR signaling in the R175H mutant cell line is consistent with the lack of IRP2 protein expression changes we observed in response to iron loading and iron chelation. IRP2 protein was also unaffected by iron excess in the TP53 null cells, as well as R248Q and H193Y-expressing mutants, which also displayed low levels of FDXR protein expression. However, R273H-expressing mutants displayed low levels of FDXR protein expression and appropriate IRP2 responsiveness, which suggests the need for further investigation as to how TP53 mutation status influences the FDXR-IRP signaling pathway.

Although both FTH1 and TFRC have established IRP-independent modes of regulation, modulation in response to changes in cellular iron levels are typically IRP-dependent. However, our results indicate cells expressing TP53 mutants increase FTH1 and TFRC protein expression in response to iron excess and restriction, respectively, despite an abrogated IRP response. Iron influx into the cell can increase free iron availability and ROS production [52]. The increased oxidative stress can then activate HMOX1 and nuclear factor erythroid 2 like 2 (NFE2L2) signaling pathways, of which FTH1 is a transcriptional target [53,54]. Thus, the observed increase in FTH1 protein expression in TP53 mutant-expressing cells could be mediated by NFE2L2 transcriptional activation. TFRC expression can be regulated independently of IRP via the binding of hypoxia inducible factors (HIFs) to its promoter region [55,56]. The prolyl hydroxylases that regulate HIF stability are iron-dependent, and thus iron chelation promotes HIF-mediated TFRC transcription [57,58]. As TFRC transcript levels were increased following DFO treatment regardless of TP53 mutation status, elevated TFRC protein expression in TP53 mutant cells could be the result of both IRP-dependent and independent modes of regulation. Future research should examine the relative contributions of HIF versus IRP-mediated control of TFRC expression in distinct TP53 mutation types.

## 5. Conclusions

Altogether, our findings illustrate that TP53 mutation status can have a significant impact on cellular iron homeostasis. Our results also highlight the importance of distinguishing between TP53 mutation subtypes, as distinct subtypes exhibit unique phenotypic responses. The frequency with which TP53 is mutated in human cancers necessitates a better understanding of how distinct TP53 mutation subtypes influences tumor cell iron metabolism. This understanding could in turn enhance chemotherapeutic strategies for targeting mutant TP53 and suggest novel chemotherapies that leverage the relationship between TP53 mutation and neoplastic iron dysregulation. 

## Supplementary Materials

The following are available online at https://www.mdpi.com/2072-6643/11/9/2144/s1, Table S1: Proteomics analysis of H1299 cells following induction of WT or R175H mutant TP53.
